# Single-Cell Analysis of Ploidy and Centrosomes Underscores the Peculiarity of Normal Hepatocytes

**DOI:** 10.1371/journal.pone.0026080

**Published:** 2011-10-12

**Authors:** Francesca Faggioli, Paolo Vezzoni, Cristina Montagna

**Affiliations:** 1 Milan Unit, Istituto di Ricerca Genetica e Biomedica, Consiglio Nazionale delle Ricerche, Milan, Italy; 2 Medical Biotechnologies Unit, Istituto Clinico Humanitas, Rozzano, Milan, Italy; 3 Departments of Genetics, Albert Einstein College of Medicine, New York, New York, United States of America; 4 Pathology, Albert Einstein College of Medicine, New York, New York, United States of America; University of Turin, Italy

## Abstract

Polyploidization is the most well recognized feature of the liver. Yet, a quantitative and behavioral analysis of centrosomes and DNA content in normal hepatocytes has been limited by the technical challenges of methods available. By using a novel approach employing FISH for chromosomes 18, X and Y we provide, for the first time, a detailed analysis of DNA copies during physiological development in the liver at single cell level. We demonstrate that aneuploidy and unbalanced DNA content in binucleated hepatocytes are common features in normal adult liver. Despite the common belief that hepatocytes contain 1, 2 or no more than 4 centrosomes, our double staining for centrosome associated proteins reveals extranumerary centrosomes in a high percentage of cells as early as 15 days of age. We show that in murine liver the period between 15 days and 1.5 months marks the transition from a prevalence of mononucleated cells to up to 75% of binucleated cells. Our data demonstrate that this timing correlates with a switch in centrosomes number. At 15 days the expected 1 or 2 centrosomes converge with several hepatocytes that contain 3 centrosomes; at 1.5 months the percentage of cells with 3 centrosomes decreases concomitantly with the increase of cells with more than 4 centrosomes. Our analysis shows that the extranumerary centrosomes emerge in concomitance with the process of binucleation and polyploidization and maintain α-tubulin nucleation activity. Finally, by integrating interphase FISH and immunofluorescent approaches, we detected an imbalance between centrosome number and DNA content in liver cells that deviates from the equilibrium expected in normal cells. We speculate that these unique features are relevant to the peculiar biological function of liver cells which are continuously challenged by stress, a condition that could predispose to genomic instability.

## Introduction

Despite the body of work investigating the mechanisms leading to liver polyploidization [1]–[4], a detailed analysis of hepatocytes at the single cell level during their physiological development has not yet been described. The literature reports that, contrary to most other cell types, adult hepatocytes are polyploid cells with a DNA content of 4, 8 or even 16 haploid genomes[1], [5]. In fetal and early neonatal life, hepatocytes are mononucleated diploid cells that, quite abruptly, become binucleated and polyploid soon after weaning [2], [6]–[7]. It is well known that the phenomenon of polyploidization includes the generation of tetraploid intermediates [8]–[9]. These cells have the potential to generate aneuploid progeny in the subsequent cell division, because of the presence of supernumerary centrosomes. Normally in diploid cells, at the beginning of mitosis, a single centrosome duplicates and the mother and daughter organelles migrate to opposite cell poles, directing the formation of the spindle, to guarantee a balanced chromosomal segregation [10]. However, supernumerary centrosomes can cluster together, acting as two single units mimicking a bipolar spindle, or as single entities that generate multipolar spindles in which chromosomes are improperly segregated into two or more daughter cells [11]. The result of a multipolar division is progeny with an unbalanced DNA content, differing in one or a few chromosomes. A general tool for the analysis of hepatocyte DNA content is the staining of nuclei upon digestion of liver tissue with propidium iodide followed by quantification of fluorescent intensity with a flow cytometer [1]. Another approach is based on the evaluation of fluorescence intensity of thin liver tissue sections stained with Hoechst 33342 using an epi-fluorescent microscope. The identity of mono- or binucleated hepatocytes is determined by comparing nuclear to membrane labelling [2]. These classical approaches lack the sensitivity to detect the small differences in DNA content that result from unbalanced chromosomal segregation. Moreover, the use of tissue sections stained with Hoechst or DAPI for the determination of DNA content incurs in several technical problems. For one, the inclusion of various cell layers makes it difficult to determine the individual cell identity, even when associated to membrane staining. Additionally, the partial amputation of nuclei could be responsible for false evaluation of the staining intensity and, consequentially, the DNA content. In addition to these technical challenges, a quantitative and behavioural analysis of extranumerary centrosomes in normal liver cells has not been thoroughly performed. Guidotti and colleagues report that *in vitro* cultured binuclear hepatocytes carry one, two or four centrosomes relative to the DNA content of each nucleus [2]. In cases where four centrosomes were observed, they clustered in pairs at opposite poles allowing for a bipolar spindle to assemble normally. Nelsen and colleagues reported similar data in normal hepatocytes; however, they detected a small fraction of normal hepatocytes with three centrosomes, while >4 centrosomes were detected only in cells transfected with cyclin D (see Figure 2B and Figure 8 in ref [12]). In the model where the two nuclei of an endoduplicated binuclear cell divide synchronously, as proposed by Guidotti and colleagues, the genesis of cells with 3 centrosomes is difficult to explain. More recently, to test the hypothesis that adult polyploid hepatocytes are able to generate progeny with halved chromosome content (a phenomenon known as “ploidy conveyor”), Duncan et al. [13] demonstrated that after 5 days in culture tetraploid hepatocytes produced daughters cells with 8n and 2n DNA content. They were the first to show that tetraploid liver cells are able to divide and that, during cell division, centrosomes can polarize multipolar spindles, although this is a temporary step followed by its reorganization into a bipolar spindle. The formation of multipolar spindles is a feature of tetraploidy and cells with extranumerary centrosomes. However, even in Duncan's paper a deep analysis of the number of centrosomes for mono and binucleated hepatocytes during different developmental stages has not been shown.

The application of interphase FISH to the analysis of hepatocytes allows for an unprecedented analysis of ploidy at the single cell level [4], [14]. This method is highly sensitive and hundreds of cells for any given sample can be analyzed. Furthermore it makes possible to distinguish binucleated hepatocytes from mononuclear cells by using a DIC filter [4]. Likewise, for a comprehensive, integrative analysis of DNA copy number and proteins involved in the mitotic process, the interphase FISH technique can be combined with additional fluorescent approaches, such as immunostaining of centrosome components (γ-tubulin and pericentrin).

In this study, in order to contribute to the understanding of the mechanisms underlying controlled polyploidy in physiologically normal cells, we investigated normal mouse hepatocytes with tools allowing the analysis of ploidy, nuclear synchronization and centrosome enumeration and function at the single cell level. We report several findings that reveal that hepatocytes are more complicated than expected.

## Materials and Methods

### Mice and cells

Mice were maintained in accordance with the guidelines from the Italian Ministry of Health. The Project was approved by the Istituto Clinico Humanitas Animal Care and Use Committee on March 10th, 2008 (number: Vezzoni 1/2008) and by Ministry of Health on April 4th, 2008. CD1 mice (18 days p.c., 15 and 21 days, 1.5, 4, 5 and 7 months old) were obtained from Charles River. To obtain fresh suspensions of juvenile and adult hepatocytes, liver perfusion was carried out as previously described [4].

### Fluorescence in situ hybridization

Fluorescence in situ hybridization (FISH) was performed using a locus specific probe for the X chromosome and painting probes for the 17, 18 and Y chromosomes. The BAC clone RP23-113K2, mapping to the distal region of the X chromosome, was obtained from the Children's Hospital, Oakland CA. This probe was labeled by nick translation using biotin-16-dUTP (Roche Diagnostic, Indianapolis, IN) and was detected by Alexa Fluor 647-conjugated streptavidin antibody (Invitrogen, Carlsbad, CA). To obtain painting chromosomes for the 17, 18 and Y chromosomes, flow sorted DNA for 17, 18 and Y chromosomes (M.A. Ferguson-Smith, University of Cambridge, Cambridge UK) were labelled by DOP-PCR with Spectrum Aqua-dUTP (Perkin Elmer, Waltham, MA) and Spectrum Orange-dUTP (Abbott Laboratories, Abbot Park, IL). Square coverslips in which hepatocytes were plated after perfusion were incubated in denaturation solution (FA/SSC) at 90°C for 1′ and 45″ and then dehydrated with serial ethanol washing steps (70 ice-cold, 90, 100% for 3′ each). Probes were denaturated in the hybridization solution (50% dextran sulfate/SSC) at 85°C for 5 min, applied onto the slides and incubated overnight at 37°C in a humidified chamber. After washing with 50% formamide/2X SSC and 1X SSC for 5′ the coverslips were incubated at 37°C with blocking solution (3% BSA). Thereafter for the detection of the locus specific probe the coverslips were incubated with the previously mentioned secondary antibody. Slides were counterstained with DAPI, dehydrated with ethanol series and mounted for imaging.

### Centrosome analysis, nucleation assay and H3 staining

After perfusion, single hepatocytes were plated in chamber slides and incubated at 37°C overnight in William's E medium (Invitrogen, Carlsbad, CA) supplemented with 15% Fetal Calf Serum (FCS) (Sigma, St Louis, MO) and antibiotics (100 U/ml penicillin and 100 µg/ml streptomycin). For centrosome visualization, cells were fixed in ice cold MeOH for 10′ rinsed 3 times with PBS 1x and incubated with goat serum 5% for 1 h at 37°C. Hepatocytes were incubated with mouse anti γ-tubulin and rabbit anti pericentrin (Abcam, Cambridge, MA 1∶500) and detected with anti mouse Alexa-488 and anti rabbit Alexa-647 (Invitrogen AlexaFluor 1∶1000). For nucleation assay, cells were incubated with Nocodazole (10 mg/mL) for 1.5 h at 37°C followed by 15′ on ice. Hepatocytes were washed with PBS 1x at room temperature and incubated with fresh medium at 37°C for 5′ to allow for α-tubulin polymerization, thus testing centrosome nucleation activity. The cells were fixed in MeOH on ice for 10′ and store at 4°C until their use. For microtubules and centrosome detection after blocking (10% goat serum, Sigma) hepatocytes were incubated with mouse α-tubulin (Abcam 1∶500), rabbit anti γ-tubulin or rabbit anti-pericentrin (Abcam 1∶500) specific antibodies. Secondary detection was performed with anti mouse 488 antibody (AlexaFluor 1∶1000) and goat anti-rabbit 647 antibody (Abcam 1∶1000). The cells were finally counterstained with DAPI. For phospho-histone H3 assay, hepatocytes were fixed in 1% PFA for 10 minutes, washed 3 times in PBS 1x and permeabilized with Triton X-100, 0.3% for 10 minutes at room temperature. After blocking with 5% goat serum (Sigma, St Louis MO), the cells were incubated with a mouse monoclonal antibody against histone H3S10P (mAbcam 14955, 1∶500) and detected with anti-mouse AlexaFluor 488 secondary antibody (1∶1000).

### Two colors combined FISH and immunofluorescence

Liver cells from a 45 day-old mouse were plated in special glass coverslips carrying a grid that allows mapping of the exact position of the cells (BELLCO, Vineland, NJ). To determine the centrosome number hepatocytes were stained with an anti γ-tubulin antibody as previously described. In the first step of our experiment images corresponding to centrosome signals were acquired with a dye specific cube together with its differential interference contrast image (DIC). The physical location of each cell was recorded with the aid of numbers and letters engraved on the grid. The second step consisted of removing the antifade and carrying out a FISH hybridization with a Y-specific painting probe to determine the ploidy (see above for the procedure). During this second step, the previously acquired DIC image was essential for recognizing the same field used for the γ-tubulin staining and used to unambiguously associate the number of centrosomes and the DNA content of each cell.

### Image acquisition

**Figure 1:**
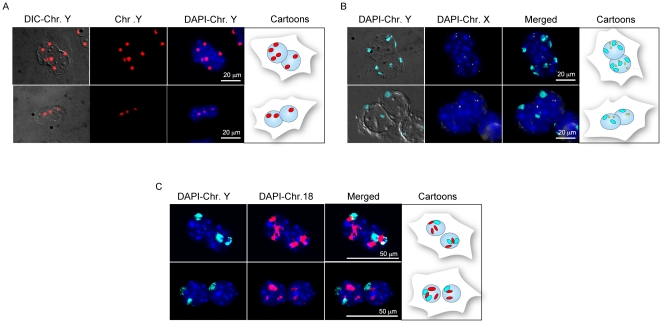
Interphase FISH of binucleated hepatocytes. (A) Representative binucleated cell with discordant DNA content between the two nuclei (8n vs. 4n, top and 2n vs. 4n, bottom) as detected by the number of copies of chromosome Y (in red). (B) Ploidy of binucleated hepatocyte analyzed with a chromosome paint for Y (cyan) and a locus specific probe for X (yellow). In each nucleus the number of copies for the Y chromosome match the expected number for the X chromosome (top 3X, 3Y and 4X, 4Y; bottom 2X, 2Y and 1X, 1Y). However, the ploidy between the two nuclei is discordant. (C) Binucleated hepatocytes with unbalanced DNA content for chromosome Y (cyan) to which corresponds aneuploidy for the autosome 18 (in red) (top: 1 Y chr. with 3 copies of 18 chr. in the left nucleus and 2 Y with 2 copies of 18 in the right nucleus; bottom: 2 Y with 3 copies of chr. 18 in the left nucleus. In this nucleus one spot for chr. 18, the top one, could also be the result of two overlapping chromosomes 18, resulting in 4 signals. In either case the left nucleus is unbalanced with respect to the right nucleus, which contains 1Y with 2 copies of 18). The cartoons on the right of each panel summarize the ploidy for each cell.

For Figures 1A and 1B interphase cells were imaged with an Olympus BX61 microscope with an UPlanSApo 40 X oil immersion lens, an Hg arc lamp for excitation and narrow band filters for all fluorescent emission and equipped with a Cooke SensicamQE camera with IPLab imaging software for image acquisition. Images of interphase cells for each slide were acquired for the Spectrum Orange, Cy5 and Spectrum Aqua dyes. An IP lab script was generated to acquire images; a DIC image was acquired first to ensure that bi-nucleated cells shared the same cytoplasm even though the hepatocytes were diluted enough to avoid high density cell plating. Multiple focal planes were acquired for each channel to ensure that signals on different focal planes were included: eight focal planes for chromosome painting and thirteen different focal planes for locus specific probes were acquired.

**Figure 2:**
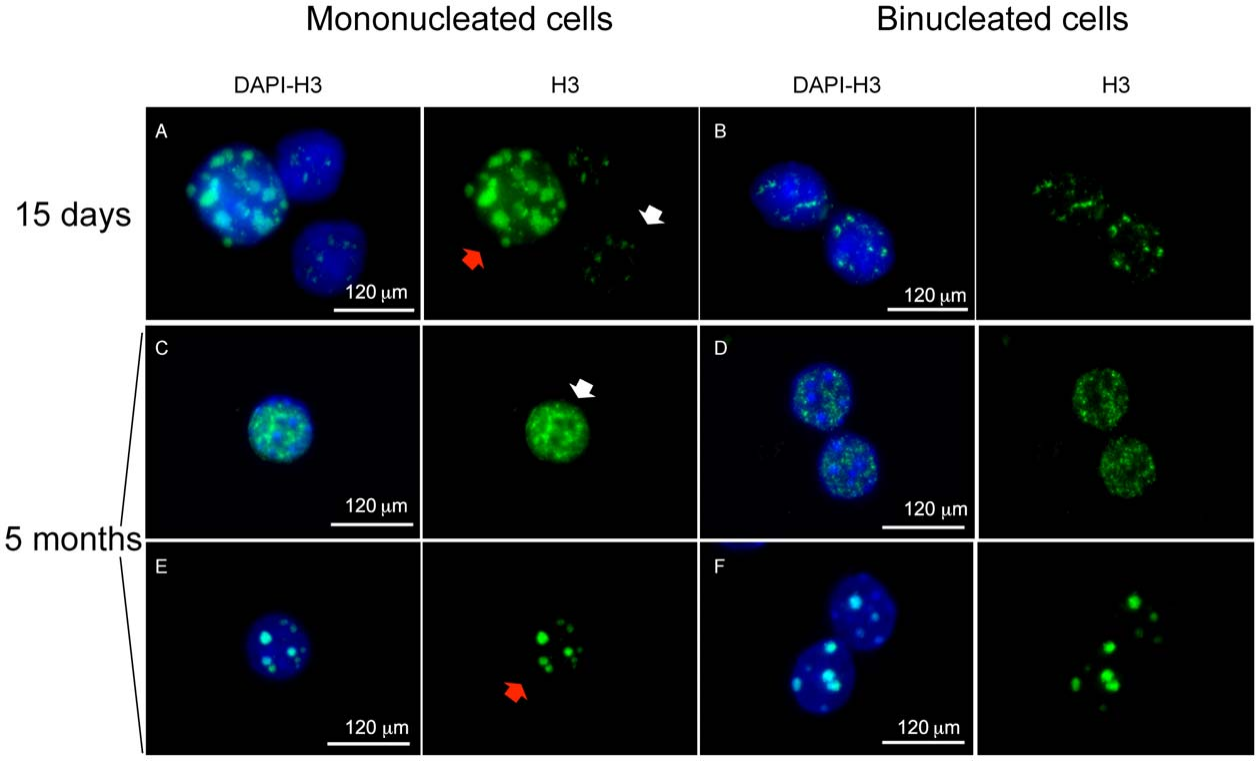
Immunofluorescence with anti-histone H3S10P antibody. (A, B) Examples of binucleated and mononucleated cells from a 15 day-old mouse. In this young mouse the mononucleated cells show different status of chromatin modification as detected by an anti phospho-histone H3 antibody (green). Small H3S10P foci or a diffuse staining (white arrows) are indicative of non replicative cells, while larger H3S10P foci at pericentric chromatin (red arrows) suggest that cells are proceeding to the G2/M phase. (C-F) Cells from a 5 month-old mouse. (B,D,F) Binucleated cells from young (B) and old (D,E) mice with small H3S10P foci (B,D) and with dot-like structures (F). The staining for both nuclei is comparable.

For Figure 2, images were acquired with a manual inverted fluorescence microscope (Axiovert 200, Zeiss) with fine focusing oil immersion lens (× 60, NA 1.35). The resulting fluorescence emissions were collected using 425-to-475 nm (for DAPI) and 500-to-550 nm (for AlexaFluor488) filters. The microscope was equipped with a Camera Hall 100 and with the Applied Spectral Imaging software.

**Figure 3:**
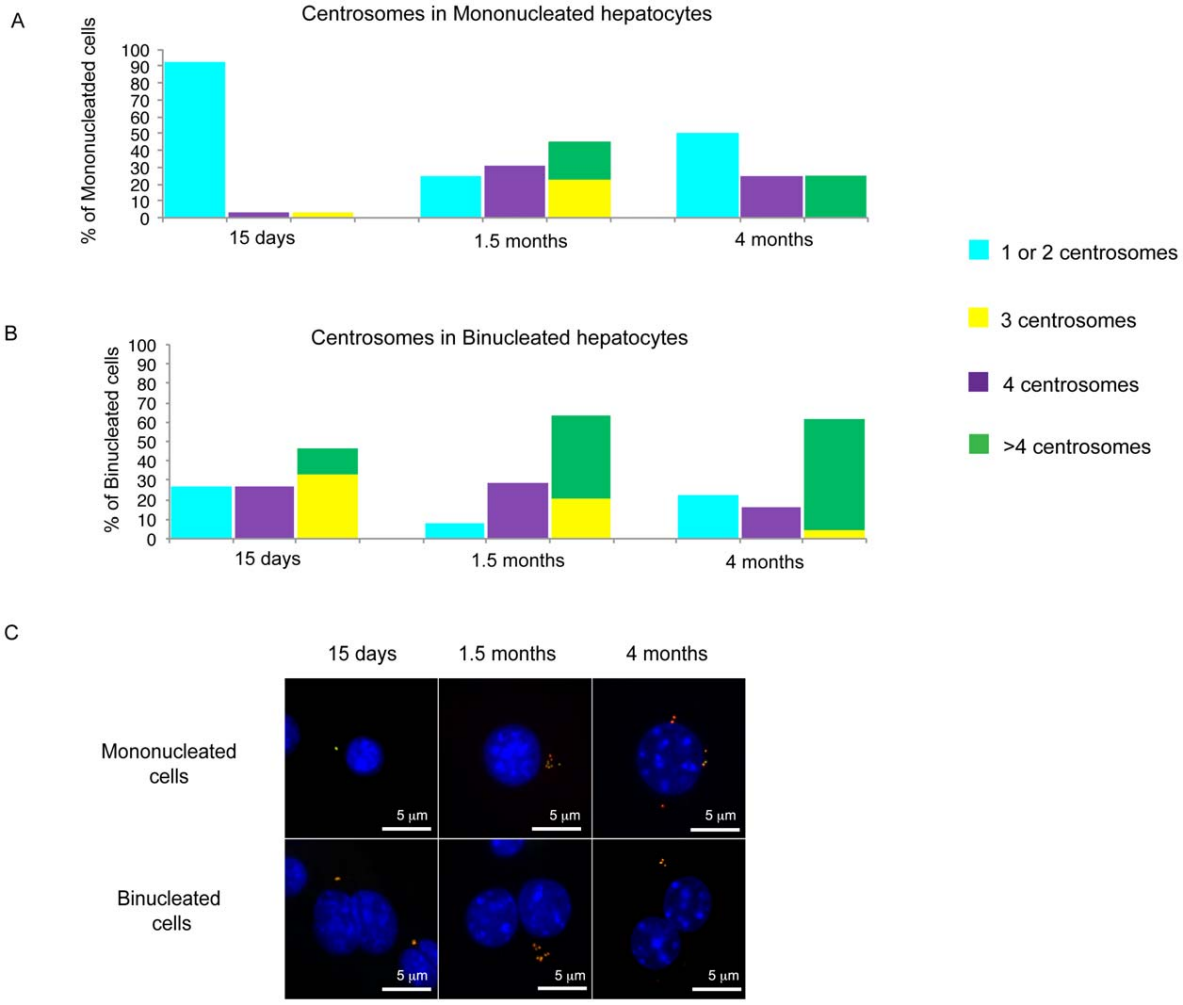
Centrosome analysis. (A, B). The plots summarize the percentage of mononucleated (A) and binucleated cells (B) classified according to the number of centrosomes found by co-immunostaining with anti γ-tubulin and anti-pericentrin. In this plotting the percentage of cells with 4 centrosomes is kept separate from cells carrying 1 and 2 centrosomes since, depending upon the DNA content, cells with 4 centrosomes could be classified as normal or abnormal. (C) Examples of normal and extranumerary centrosomes detected by colocalization of γ-tubulin (green) and pericentrin (red) in mono (top) and binucleated (bottom) hepatocytes for the different ages analyzed. For the older mice analyzed some example of extra-centrosomes are reported.

**Figure 4:**
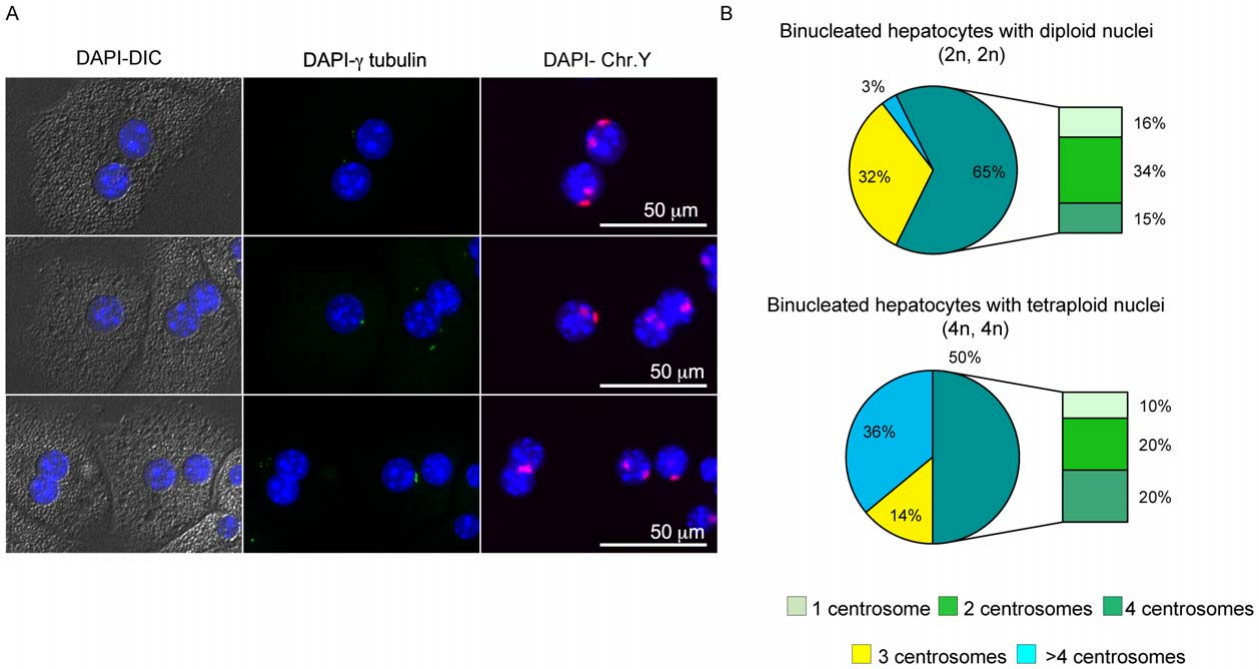
Analysis of centrosomes in relation to the DNA content. Panel (A) Examples of hepatocytes analyzed by combined FISH (chromosome Y red) and immunofluorescence for γ-tubulin (green). A binucleated hepatocyte with two tetraploid nuclei as detected by copies of the Y chromosome carries only three centrosomes (top). A tetraploid mononucleated cell with one centrosome (abnormal condition) is shown in the middle panel together with a binucleated tetraploid cell with 4 centrosomes. We note that the centrosomes are positioned asymmetrically. A binucleated hepatocyte with six centrosomes is shown in the bottom panel. (B) The top pie summarizes the percentage of binucleated hepatocytes with both diploid nuclei showing the distribution of centrosomes (1, 2, 3, 4 and >4); on the bottom the centrosome distribution is plotted for binucleated cells with tetraploid nuclei.

**Figure 5:**
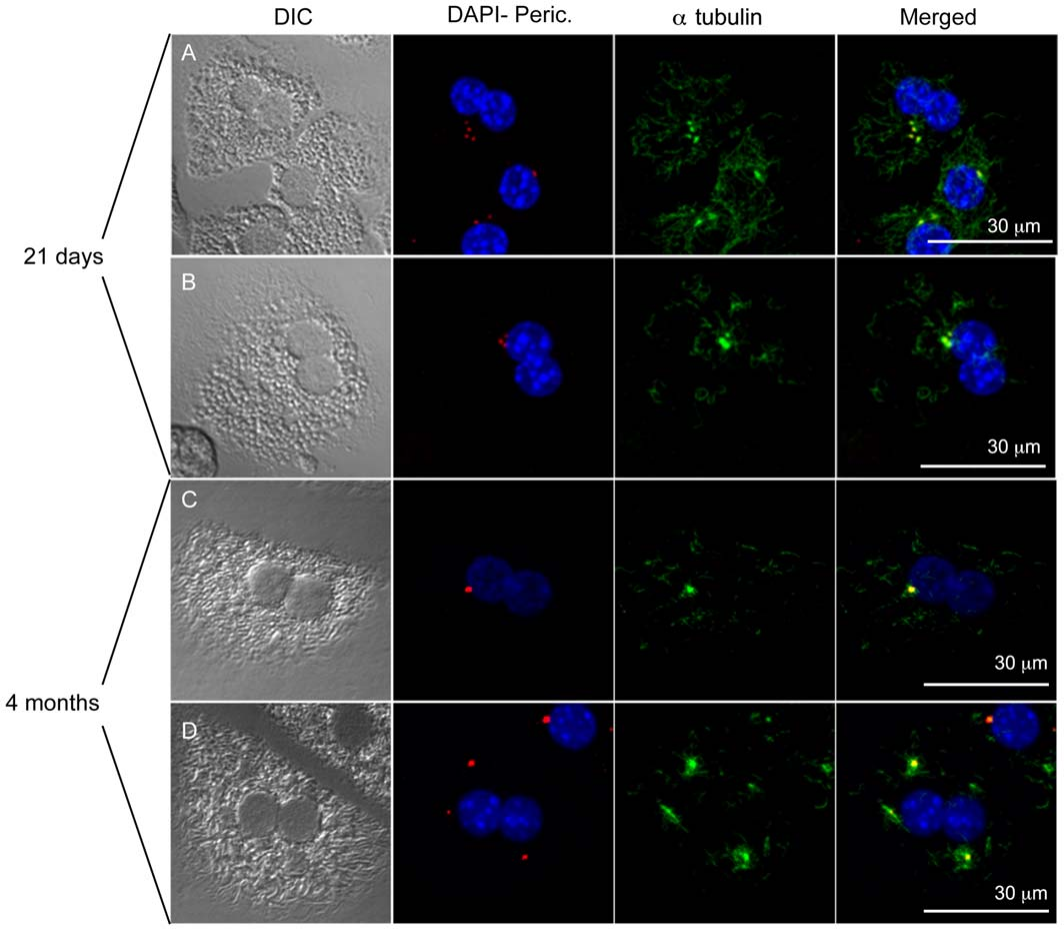
Nucleation assay. (A, B) Examples of one mono and two binucleated cells in a 15 day- old mouse with normal and abnormal centrosomes number. All centrosomes maintain the ability to polymerase tubulin, even the binucleated cell in B with three centrosomes. (C, D) In 5 month- old mice binucleated cells with normal (1, as shown in C) and abnormal (3, as shown in D) centrosomes the nucleation assay suggests that all centrosomes are potentially active even at 4 months of age when cells are prevalently in the Go phase.

For Figures 1C, 3 and 4, specimens were acquired using a motorized inverted fluorescence microscope, CellR (Olympus) also equipped with DIC. FISH images were acquired with fine focusing oil immersion lens (× 60, NA 1.35 and × 40) in optical sections of 0.5 µm. The resulting fluorescence emissions were collected using 425-to-475 nm (for DAPI), 430-to-450 nm (for Spectrum Aqua), 565-to-615 nm (for Spectrum Orange), 500-to-550 nm (for AlexaFluor488) and 655–750 nm (for AlexaFluor647) band-pass filters. The microscope was equipped with a CCD Olympus Fluo View camera. For Figure 5, cells were acquired with fine focusing oil immersion lens (× 40) in optical sections of 0.5 µm using an FV1000 laser scanning confocal microscope (Olympus) equipped with a FV1000 software, and operating in channel mode with 405, 488 and 633 nm excitations. The resulting fluorescence emissions were collected using 425-to-475 nm (for DAPI), 500-to-550 nm (for AlexaFluor488) and 655-to-750 nm (for AlexaFluor647) band-pass filters; DIC was also used. The z stacks were acquired with resolution of 1 Airy unit to allow three-dimensional reconstructions. Images were analyzed with tools available through ImageJ (http://rsb.info.nih.gov/ij/) and Photoshop (Adobe).

## Results

### Some binucleated hepatocytes show an unbalanced chromosome content

Fetal and neonatal mouse hepatocytes are diploid cells. Polyploidy usually starts at the second/third week of age, in concomitance with weaning, although the appearance of binucleation and polyploidy can change according to the mouse strain examined [1], [5]–[6]. To confirm that this also applies to the strain we used in this study (CD1), we analyzed the frequency of binucleated and mononucleated cells with classical haematoxylin/eosin staining at different mouse ages. We performed our analysis on two CD1 mice of four different ages: fetal (18 days post coitum), before weaning (15 days), young (1.5 months) and adult (3 months) mice.

Our results show that CD1 hepatocytes are mainly mononucleated cells in fetal and perinatal life, but become binucleated at 15 days post partum. At 1.5 months most hepatocytes become binucleated cells and their percentage does not vary appreciably later in life (Figure S1).

As discussed above, the previous methods used for the evaluation of the DNA ploidy of individual nuclei, either in monucleated or binucleated hepatocytes, cannot be considered trustworthy because of the technical challenges. We and others have recently used chromosome specific probes to investigate the chromosome content of liver cells [4], [15]. As stated before, in binucleated liver cells, it is often assumed that the chromosome content is similar in both nuclei and that binuclear cells divide synchronously into two mononuclear 4n hepatocytes [2]. However, this aspect needs further investigation, since data to support this hypothesis were obtained by *in vitro* cultures, which might not represent the physiological processes taking place in normal liver. Moreover, the data were generated in young (28 days old) rats, at a time in which liver cells are still actively dividing and start to become polyploid.

To better investigate whether the two nuclei of binucleated cells actually have the same chromosome content, we performed an interphase FISH experiment on single hepatocytes of 7 months old mice, using Y and X chromosome specific probes as previously described [4]. We observed that, based on the ploidy of sex chromosomes, most binucleated cells from mature livers (7 months old) carry nuclei with the same chromosomal content (2n, 4n or even 8n). However, in two different experiments we found that 8.7% of binucleated hepatocytes showed a discordant number of Y chromosomes between the two nuclei, when analysed only with the Y chromosome painting probe (Figure 1A). In addition, a similar percentage (8%) of cells with an unbalanced DNA content in the two nuclei but with a perfect match of the signals in each nucleus (discordant nuclei for ploidy) was found when the Y chromosome painting probe was used in combination with a BAC X-chromosome specific probe (Figure 1B). Our observations suggest the presence of binuclear hepatocytes with unbalanced DNA ploidy between the two nuclei at least for the sex chromosomes in the adult mice.

To investigate whether also the autosome ploidy was unbalanced in the time points analyzed with haematoxylin/eosin staining, we performed additional FISH experiments by combining paint probes detecting the Y chromosome with a probe for either the 17 or 18 autosome. In cells from fetal livers and in 15 day-old mice, all signals were compatible with a diploid content for both nuclei (Y and 17 probes used, data not showed), while in a 4 month-old mouse we found that some binucleated cells showed unbalanced signals for Y and 18 chromosomes between the two nuclei (Figure 1C). Altogether, the entity of the phenomenon described above is in the order of 21% (18/87 cells); however, since our analysis targeted only a few chromosomes, the aneuploidy rate could be higher.

### Nuclei of binucleated hepatocytes are mostly synchronous

To test the hypothesis that the unbalanced DNA content in binucleated hepatocytes is the result of asynchronous cell division within the same cell, we visualized in a 15 day- and a 5 month-old mouse the level of chromatin modification through immunostaining with anti phosho-histone H3. The phosphorylation of H3 at serine 10 occurs during interphase and mitosis. In interphase the phosphorylation of H3 affects only a subset of genes, correlates to their transcriptional activation and appears as small H3SP10 foci. However, in late G2 phase the phosphorylation of H3 occurs also in pericentromeric heterochromatin. At the G2/M stage thus an anti-H3S10P antibody is visible as a dot-like structure and is indicative of active replication. This is consistent with a role of “mitotic marker” attributed to H3S10P. In mice from both age, mononucleated cells showed a variety of dot-like structures along with a more diffuse or weak staining suggestive of cells in different stages of the cell cycle (Figure 2A, C, E). A similar situation was found for binucleated hepatocytes (Figure 2B, D, F), but the pattern of staining between the two nuclei was usually identical. This observation suggests that binucleated cells nuclei are synchronous, as suggested by Guidotti et al [2]. However, we cannot exclude the possibility of fusion of G1–S cells or G2–S cells that rest in S phase upon synchronization and subsequently proceed throught the next phase of the cell cycle, as shown by Wong and Stearns [16]. We can also speculate that with our approach we can detect only the final step of the phenomenon. Based on this consideration, the use of an *in vitro* system of cell fusion would represent an additional approach to monitor this process and establish how synchronous cells evolve.

### Normal hepatocytes bear extranumerary centrosomes

The centrosome is an organelle that serves as the main microtubule organizing centre as well as the regulator of cell cycle progression. The majority of diploid cells contain either one or two centrosomes, depending on their phase within the cell cycle. In order to correlate centrosome number with the occurrence of polyploidy and aneuploidy and to test whether extranumerary centrosomes might have functional consequences on polyploidization, we tested centrosomes behaviour in mouse hepatocytes by enumerating and performing functional analysis at different ages. To provide strict controls for the entire procedure, hepatocytes obtained after liver perfusion were analyzed using two distinct antibodies against γ-tubulin and pericentrin, that both recognize the centrosome structure, and for this reason colocalize.

We found a clear relationship between ploidy and centrosome number. Diploid hepatocytes from young mice contained one or two centrosome(s) as expected, however adult hepatocytes, concomitantly with switching in ploidy, contained variable numbers of centrosomes. In tetraploid and octaploid cells, 2, 4 or an even number of centrosomes is expected. Cells with these numbers were indeed present in the adult liver and were accounted as normal. On the other hand, we also detected a large fraction of cells with an unexpected centrosome number (3 or more than 4, see Table 1). This percentage increases with the age of the mice, since no abnormal centrosome distribution was seen in fetal liver cells. At 15 and 1.5 months of age, however, 11/122 (9%) and 71/123 (58%) of hepatocytes had an abnormal centrosome count (either 3 or >4) respectively. Therefore, the level of centrosome abnormalities correlates with the changes in ploidy occurring with mouse aging. At 4 months of age, 35/56 cells (62,5%) had an abnormal centrosome number, indicating that at around 2 months of age a plateau is reached. Interestingly, binucleated liver cells with 3 centrosomes seem to be restricted to specific liver development times, since we identified a high percentage of 3-centrosome cells concomitantly with the appearance of binucleated cells at 15 days, and this rate declines progressively until 4 months of age, at which stage cells with more than 4 centrosomes predominate (Figure 3A, B). Figure 3C shows examples of mono and binucleated hepatocytes with a normal number of centrosomes for the 15-day mice analyzed and an abnormal number of centrosomes for the older ages analyzed.

**Table 1:** Percentage of mononucleated and binucleated hepatocytes with abnormal centrosome numbers in mice of different ages.

Mouse age	Mononucleated cells				Binucleated cells			
	Examined Cells	N.centr = 1,2	N.centr = 4	N.centr = 3, >4	Examined Cells	N.centr = 1,2	N.centr = 4	N.centr = 3, >4
FETUS (n = 80)	80	80	0	0	0*	0	0	0
15 days (n = 122)	107	99	4	4	15	4	4	7
1.5 months (n = 123)	36	9	11	16	87	7	25	55
4 months (n = 56)	12	4	0	8	44	10	7	27

We next attempted to directly correlate centrosome numbers to the DNA content by simultaneously detecting ploidy by FISH and centrosomes by immunofluorescence. To this end, liver cells from a 45 day-old mouse were plated in glass coverslips with an enumerated grid that allows the identification of the localization of the cells. The cells were stained with anti-γ-tubulin antibody and then hybridized for the detection of chromosome Y in a combined experiment (see materials and methods for detail). Examples of the obtained results are shown in Figure 4A.

On a sample of 147 binucleated cells, we found that 65 showed a diploid content for each nucleus, 50 had a tetraploid content, only 1 an octaploid content and 3 had a triploid content; in the remaining cells, the DNA content was non concordant or technically difficult to assess. We confirmed that out of 65 binucleated cells with tetraploid content (two diploid nuclei) analyzed, a large proportion (35%) had an abnormal centrosome number (Figure 4B), in agreement with the results reported above. Interestingly, the majority of cells with abnormal centrosomes was represented by binucleated cells with 3 centrosomes (32%). On the other end, an octaploid content (two tetraploid nuclei) was more often associated to a highest number of centrosomes (36% of these cells had more than 4 centrosomes). The same trend was also seen in mononucleated cells (n = 41, Figure S2), although the number of examined cells for the mononucleated hepatocytes was too small for statistical relevance.

### Extranumerary centrosomes maintain nucleation capacity

It is well known that most mature hepatocytes are quiescent cells. Our findings regarding the presence of extra-centrosomes in adult hepatocytes prompted us to investigate their role in cell physiology. Supernumerary centrosomes are actually a specific peculiarity of certain tumour cells, and even in a high proliferative context it has been shown that not all these centrosomes maintain their ability to nucleate microtubules [17]. Therefore we investigated whether the same occurs in normal hepatocytes. We performed this analysis on 21 day- and 4 month-old mice. In the young mice, in which most cells are diploid, all centrosomes showed the ability to nucleate α-tubulin, as shown in Figure 5. Interestingly, binucleated hepatocytes with 3 centrosomes do not show functional clustering (Figure 5, row 2) since the organelles seem to be distinct entities that preserve the ability to polymerize microtubules independently. The results on adult mice (4 months) showed that most centrosomes in normal hepatocytes are still potentially active and able to nucleate microtubules, even though we expect these cells to be in a quiescent state. (Figure 5 rows 3 and 4). Therefore centrosome “inactivation” does not appear to be at work in normal polyploid liver cells.

## Discussion

Recent cytogenetic advancements have allowed the analysis of hepatocytes at the single cell level. Although polyploidy has long been recognized to occur in liver, hepatocytes have usually been investigated at the level of cell pools by cytofluorescent methods. In the present work we used a combination of cytogenetics and immunofluorescence techniques to investigate key aspects of the behaviour of these cells that can contribute to a better understanding of their biology.

Hepatocytes are peculiar mononucleated or binucleated cells with polyploid DNA content. Polyploidy, although present in some normal cells such the osteoclasts [18]–[19], is often considered a predisposition to aneuploidy, genome instability and neoplastic transformation [11], [20]–[23]. We attempted to investigate whether aneuploidy occurs in hepatocytes by using probes from the sex chromosomes and two autosomes (MMU 17 and 18). We obtained unambiguous images of unbalanced chromosome content in several cells, although the exact percentage cannot easily be determined due to the high complexity of ploidy observed in these cells. This is in agreement with recent data reported by Duncan and colleagues who showed that aneuploidy occurred in their specific, partially artificial *in vitro* setting through karyotype analysis [13]. Our data support the occurrence of aneuploidy in a completely normal setting, suggesting that the continuous duplication and/or the fusion process facilitate the acquisition of an unbalanced genome content. Since most mature hepatocytes are terminally differentiated cells, the presence of aneuploidy might not be dangerous to the organism if mechanisms of strict control are implemented. In this regard, the recent report of multiple benign tumors occurring only in liver in autophagy-deficient mice is particularly intriguing [24].

The use of interphase FISH allows for a more precise quantification of DNA content of liver cells. We were able to demonstrate that, even though the majority of hepatocytes had balanced DNA content, as reported by Guidotti et al. [2], a high rate of binucleated cells have a discordant chromosome number between the two nuclei. This finding suggests either that they originate by fusion of two cells with different DNA content or that the two nuclei have the ability to divide asynchronously. The latter hypothesis is not supported by the H3 histone assay performed in young and adult mice in the present study. In this regard, Wong and Stearns showed that the number of centrosomes of fused cells is strongly related to their phase in the cell cycle, since fusion of a G1-phase to an S-phase cell results in generation of cells with three centrosomes [16]. It is intriguing that we found the presence of binucleated cells with 3 centrosomes as a recurrent motive during the polyploidization process in mono- as well as in binucleated hepatocytes. The possibility of fusion, previously suggested by Faggioli et al [4] on the basis of chimeric binucleated cells with different patterns of sex chromosomes would explain the frequency of cells with three centrosomes. Whatever the reason for the presence of 3 centrosomes in binucleated cells, this seems to be associated with a diploid content of both nuclei (tetraploid cell). This finding, together with the increase in the centrosome number (>4) and ploidy occurring with age (see text and Figure 2A, B and Figure 3B), suggests that the 3-centrosome stage represents an intermediate step in the progression of the hepatocyte toward a full mature phenotype.

The concomitant presence of apparent abnormalities in centrosome number is an additional conundrum in the biology of hepatocytes. How a normal division could occur in presence of this abnormality is unclear since most centrosomes appear to be able to nucleate microtubules and direct spindle formation. Alternatively, cells with a high number of centrosomes could represent terminally differentiated senescent cells that would not further divide and that centrosomes are the relict of previous mitoses. Alternatively, the possibility remains that they play a different, undiscovered role in hepatocytes biology or function you can't simply put hepatocytes. Centrosomes are involved in cilia and flagella formation, and in some specialized cells hundreds of basal bodies are formed [10]. Centrosome proteins in liver cells could mediate cell-cell interaction and a high centrosome number could play a role in the adhesion of these large cells. Although this is highly speculative, it is noteworthy that polycystic kidney patients, who have structural abnormalities of primary cilia, have additional defects in other organs including the liver [25]. Very recently, it has been found that Joubert syndrome and related disorders, whose clinical picture includes liver fibrosis, is due to a defect in the TMEM216 gene (a protein involved in ciliogenesis and centrosomal docking) [26].

In conclusion, in this study we have documented, at the single cell level, the presence of unexpected features of hepatocytes. These could be related to the peculiar function of liver cells, which are continuously challenged by stress events that could require high gene expression of particular loci. This might lead to polyploidy arising by either endoduplication or fusion and to centrosome amplification, both features predisposing to genomic instability. Further studies, at the molecular level, to elucidate how the hepatocyte manages to avoid neoplastic transformation are needed to better understand the biology of this cell that is routinely exposed to high levels of potential carcinogens.

## Supporting Information

Figure S1 **This graph summarizes the percentage of mono (blue bars) and binucleated (red bars) hepatocytes at different mouse ages as analyzed by classical eosin/hematoxilin staining.**
(TIF)

Figure S2 **Analysis of centrosomes in relation to the DNA content of mononucleated hepatocytes.** The pies summarize the distribution of the number of centrosomes in the groups of mononucleated hepatocytes with diploid (A), tetraploid (B) and octaploid (C) DNA content determined on the basis of the chromosome Y signals. (D) Summary table of data plotted in A, B and C.
(TIF)
