# Supplementary figures and images for: Single-Cell Analysis of Ploidy and Centrosomes Underscores the Peculiarity of Normal Hepatocytes
Source: PLoS One. 2011 Oct 12;6(10):e26080. doi: 10.1371/journal.pone.0026080 (PMC3192148; doi:10.1371/journal.pone.0026080)

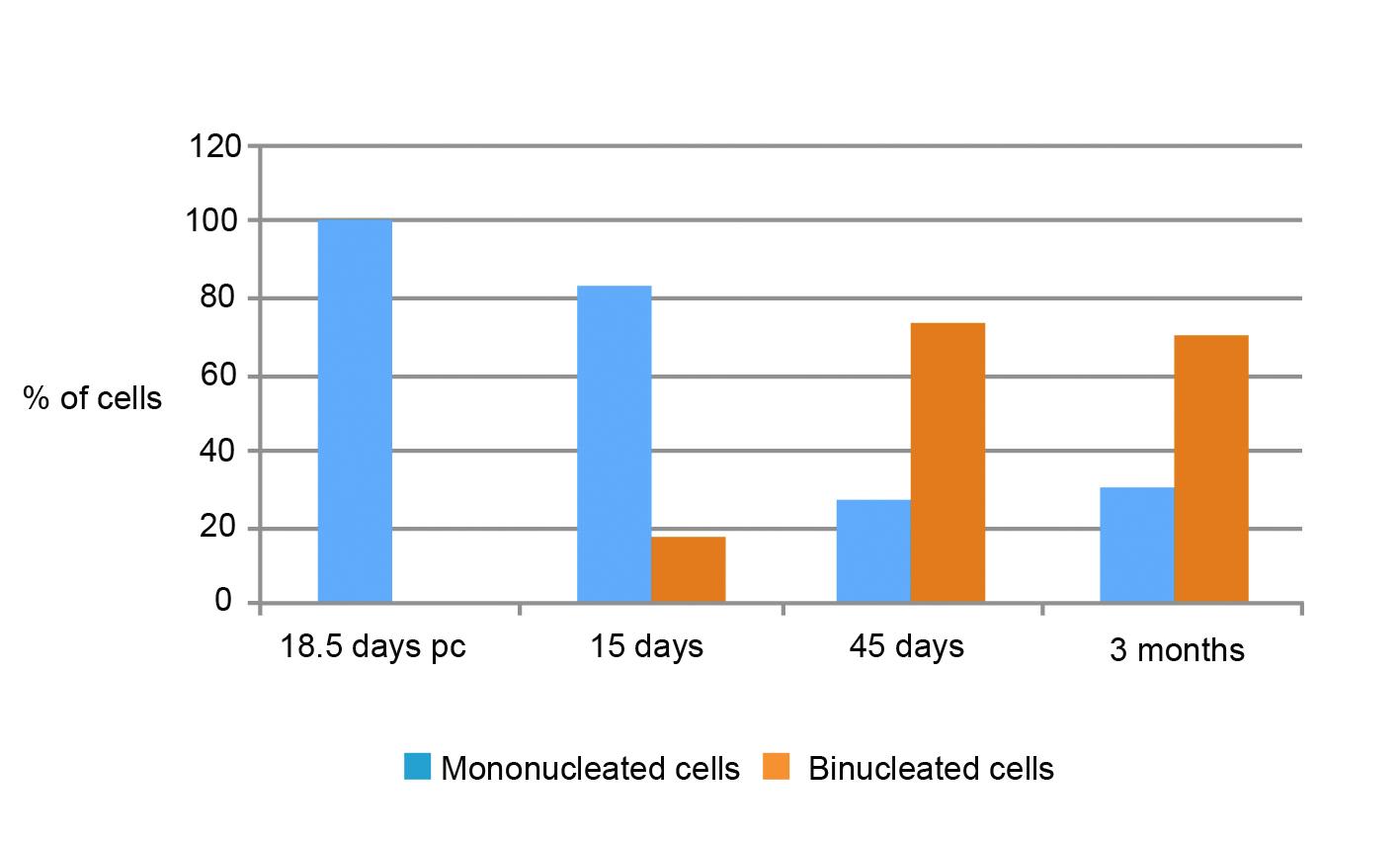

Supplement: Figure S1 — This graph summarizes the percentage of mono (blue bars) and binucleated (red bars) hepatocytes at different mouse ages as analyzed by classical eosin/hematoxilin staining. (TIF) [file pone.0026080.s001.tif]

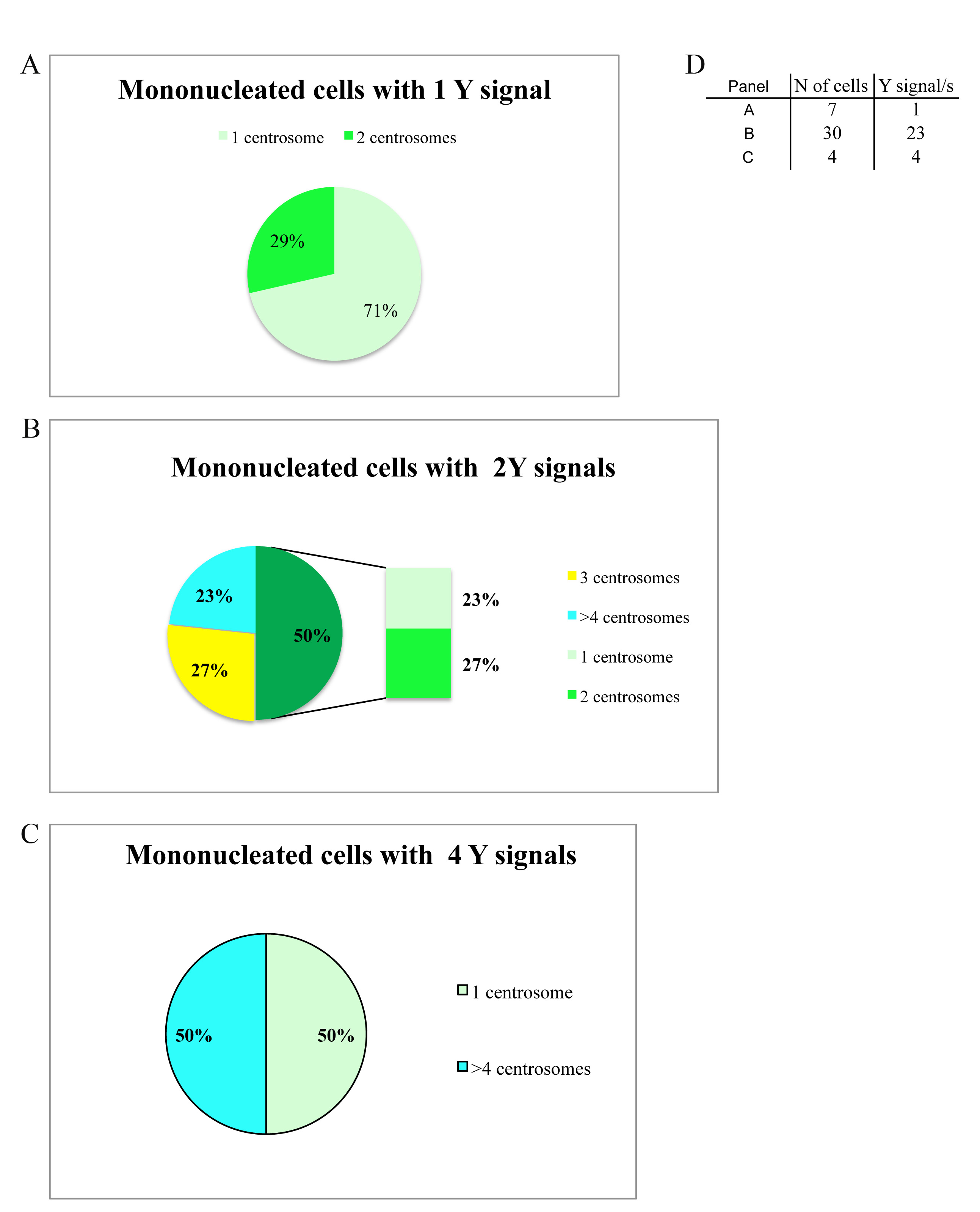

Supplement: Figure S2 — Analysis of centrosomes in relation to the DNA content of mononucleated hepatocytes. The pies summarize the distribution of the number of centrosomes in the groups of mononucleated hepatocytes with diploid (A), tetraploid (B) and octaploid (C) DNA content determined on the basis of the chromosome Y signals. (D) Summary table of data plotted in A, B and C. (TIF) [file pone.0026080.s002.tif]
